# Prostate Cancer Diagnosis Rates among Insured Men with and without HIV in South Africa: A Cohort Study

**DOI:** 10.1158/1055-9965.EPI-24-0137

**Published:** 2024-05-07

**Authors:** Yann Ruffieux, Nathalie V. Fernández Villalobos, Christiane Didden, Andreas D. Haas, Chido Chinogurei, Morna Cornell, Matthias Egger, Gary Maartens, Naomi Folb, Eliane Rohner

**Affiliations:** 1 Institute of Social and Preventive Medicine, University of Bern, Bern, Switzerland.; 2 Department of Sociology, Ludwig-Maximilians-Universität Munich, Munich, Germany.; 3 Centre for Infectious Disease Epidemiology and Research, School of Public Health, University of Cape Town, Cape Town, South Africa.; 4 Population Health Sciences, Bristol Medical School, University of Bristol, Bristol, United Kingdom.; 5 Division of Clinical Pharmacology, Department of Medicine, University of Cape Town, Cape Town, South Africa.; 6 Wellcome Centre for Infectious Diseases Research in Africa, Institute of Infectious Disease and Molecular Medicine, University of Cape Town, Cape Town, South Africa.; 7 Medscheme, Cape Town, South Africa.

## Abstract

**Background:**

Several studies have found lower prostate cancer diagnosis rates among men with human immunodeficiency virus (HIV; MWH) than men without HIV but reasons for this finding remain unclear.

**Methods:**

We used claims data from a South African private medical insurance scheme (July 2017– July 2020) to assess prostate cancer diagnosis rates among men aged ≥ 18 years with and without HIV. Using flexible parametric survival models, we estimated hazard ratios (HR) for the association between HIV and incident prostate cancer diagnoses. We accounted for potential confounding by age, population group, and sexually transmitted infections (confounder-adjusted model) and additionally for potential mediation by prostatitis diagnoses, prostate-specific antigen testing, and prostate biopsies (fully adjusted model).

**Results:**

We included 288,194 men, of whom 20,074 (7%) were living with HIV. Prostate cancer was diagnosed in 1,614 men without HIV (median age at diagnosis: 67 years) and in 82 MWH (median age at diagnosis: 60 years). In the unadjusted analysis, prostate cancer diagnosis rates were 35% lower among MWH than men without HIV [HR, 0.65; 95% confidence interval (CI), 0.52–0.82]. However, this association was no longer evident in the confounder-adjusted model (HR, 1.03; 95% CI, 0.82–1.30) or in the fully adjusted model (HR, 1.14; 95% CI, 0.91–1.44).

**Conclusions:**

When accounting for potential confounders and mediators, our analysis found no evidence of lower prostate cancer diagnosis rates among MWH than men without HIV in South Africa.

**Impact:**

Our results do not support the hypothesis that HIV decreases the risk of prostate cancer.

## Introduction

Prostate cancer is the second most common cancer among men worldwide and strongly associated with old age (1, 2). As the lifespan of people with human immunodeficiency virus (HIV) has increased since the introduction of antiretroviral therapy (ART), prostate cancer is expected to emerge as one of the most frequent cancer diagnoses among men with HIV (MWH; ref. 3). Several studies have suggested that MWH are at lower risk of being diagnosed with prostate cancer than men without HIV (1, 4, 5). The reasons for this finding are not well understood. The higher prevalence of hypogonadism among MWH, a potential protective effect of ART, or differences in prostate cancer screening practices by HIV status may play a role (1). A study in the United States found less-frequent prostate-specific antigen (PSA) testing and prostate biopsies among MWH than men without HIV (6). When accounting for these differences, prostate cancer rates did not differ by HIV status (6). Data on prostate cancer rates and screening practices by HIV status in South Africa, where a large proportion of MWH live, are scarce. A case–control study from Soweto, South Africa, found a higher HIV prevalence among men with prostate cancer than their cancer-free peers (7).

US-based studies showed that African Americans were about twice as likely as white men to develop and die from prostate cancer (8, 9). In South Africa, black African men were found to have higher odds of advanced disease than other population groups (10). Differences in prostate cancer screening practices and healthcare access may partly explain these disparities but are unlikely to fully account for the observed differences in prostate cancer incidence (11). Although prostate cancer shows high heritability, the role of genetic predisposition in the observed ethnic differences remains controversial (12, 13). Prostatitis and sexually transmitted infections (STI) have been discussed as potential risk factors for prostate cancer but the evidence remains inconclusive (14, 15).

We estimated the effect of HIV on incident prostate cancer diagnosis, taking into account confounding factors and differences in PSA testing, prostate biopsy, and prostatitis rates between MWH and men without HIV in South Africa.

## Materials and Methods

### Study design and data source

We performed a cohort study using inpatient and outpatient reimbursement claims data from a medical insurance scheme in South Africa from 2017 to 2020. This was an observational study and no randomization was performed. Of note, only about 15% of the South African population have health insurance (16). The study period was chosen based on the availability of laboratory information on PSA. Claims data were coded according to the International Classification of Diseases (ICD)-10, the Anatomical Therapeutic Chemical (ATC) Classification System, the Current Procedural Terminology (CPT), and the National Reference Price List (NRPL). We considered medical claims data available from 2011 in our analysis. The Human Research Ethics Committee of the University of Cape Town and the Ethics Committee of the Canton of Bern granted permission to analyze these data.

### Inclusion criteria and definitions

**Figure 1.**
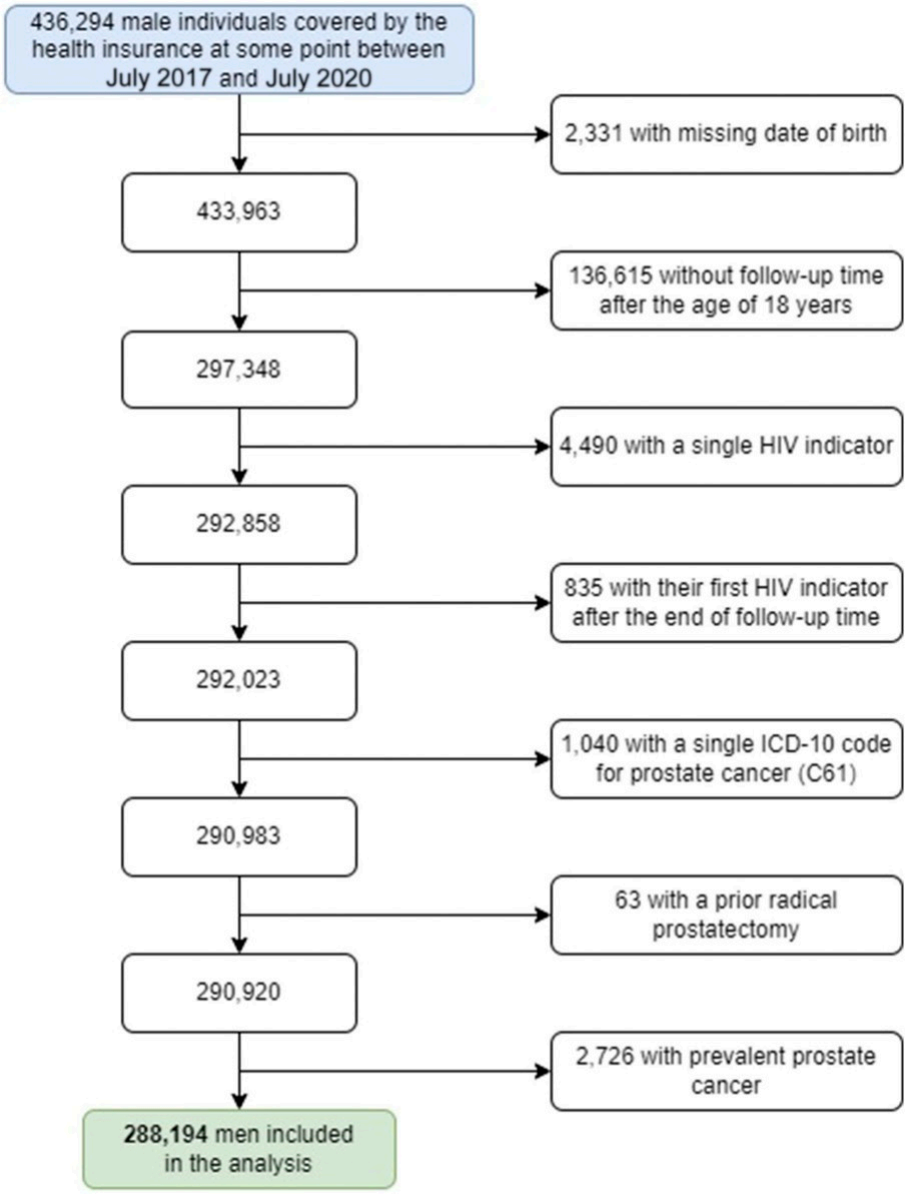
Selection of study population. The flow diagram shows the number of individuals who were excluded from the analysis and the reasons for exclusion.

We included men aged ≥18 years and covered by the medical insurance scheme at some point between July 1, 2017 and July 1, 2020. Individuals with missing information on age were excluded. Further exclusion criteria are detailed in Fig. 1. We used the following HIV indicators to identify MWH: ATC codes for ART (excluding drugs used in pre- or post-exposure prophylaxis), HIV-related ICD-10 diagnoses (B20-24, F02.4, R75, Z21), HIV-related laboratory tests (positive HIV test, HIV RNA viral load, CD4 cell count), and enrolment in the Aid for AIDS (AfA) disease management program. We regarded men with ≥2 HIV indicators as MWH and men without HIV indicator as HIV-negative. We excluded men with only one HIV indicator. We identified PSA testing from laboratory records, ICD-10 diagnoses (Z12.5), CPT codes (84512, 84153, 84154), and NRPL codes (4519). We identified prostate biopsies from CPT codes (55700, 55705, 55706) and NRPL codes (2235, 2237). Additional diagnoses of interest included prostatitis (ICD-10: N41.0-9, N51.0) and STIs (ICD-10: A51-A64). Men without ICD-10 codes for prostatitis or STIs were assumed to have no history of these diseases. Evidence of radical prostatectomy was defined based on NRPL codes (2253, 2259) and CPT codes (55840, 55842, 55845, 55866). We considered PSA test results >4 ng/mL as elevated (17).

Our main endpoint of interest were incident prostate cancer diagnoses, which we defined as ≥2 ICD-10 codes for prostate cancer (C61) on separate days. We considered the date of the first C61 code as the diagnosis date. We excluded men with a single C61 code to reduce the chance of a false-positive diagnosis. We assessed PSA testing, prostate biopsies, and prostatitis as additional outcomes.

For men without HIV, time-at-risk started at enrolment into the insurance scheme, their 18^th^ birthday, or July 1, 2017, whichever occurred last. For MWH, time-at-risk started at the date of their first HIV indicator, their 18^th^ birthday, or July 1, 2017, whichever occurred last. For all men, time-at-risk ended at the earliest of prostate cancer diagnosis, radical prostatectomy, transfer from the insurance scheme, death, or database closure (July 1, 2020). Radical prostatectomies recorded within 60 days of prostate cancer diagnosis were assumed to be linked to the cancer diagnosis and were ignored. When analyzing PSA testing rates, an individual’s time-at-risk was further right-censored at their first prostate biopsy, because PSA tests following a biopsy were assumed to be for monitoring purposes. Time-at-risk in the analysis of biopsies among men with elevated PSA was further left-truncated at the first elevated PSA test. We assumed that the PSA level remained elevated until a PSA test showed a result of <4 ng/mL, at which time point right-censoring occurred. When analyzing incident prostatitis diagnoses, time-at-risk was right-censored at the time of the first prostatitis diagnosis.

**Figure 2.**
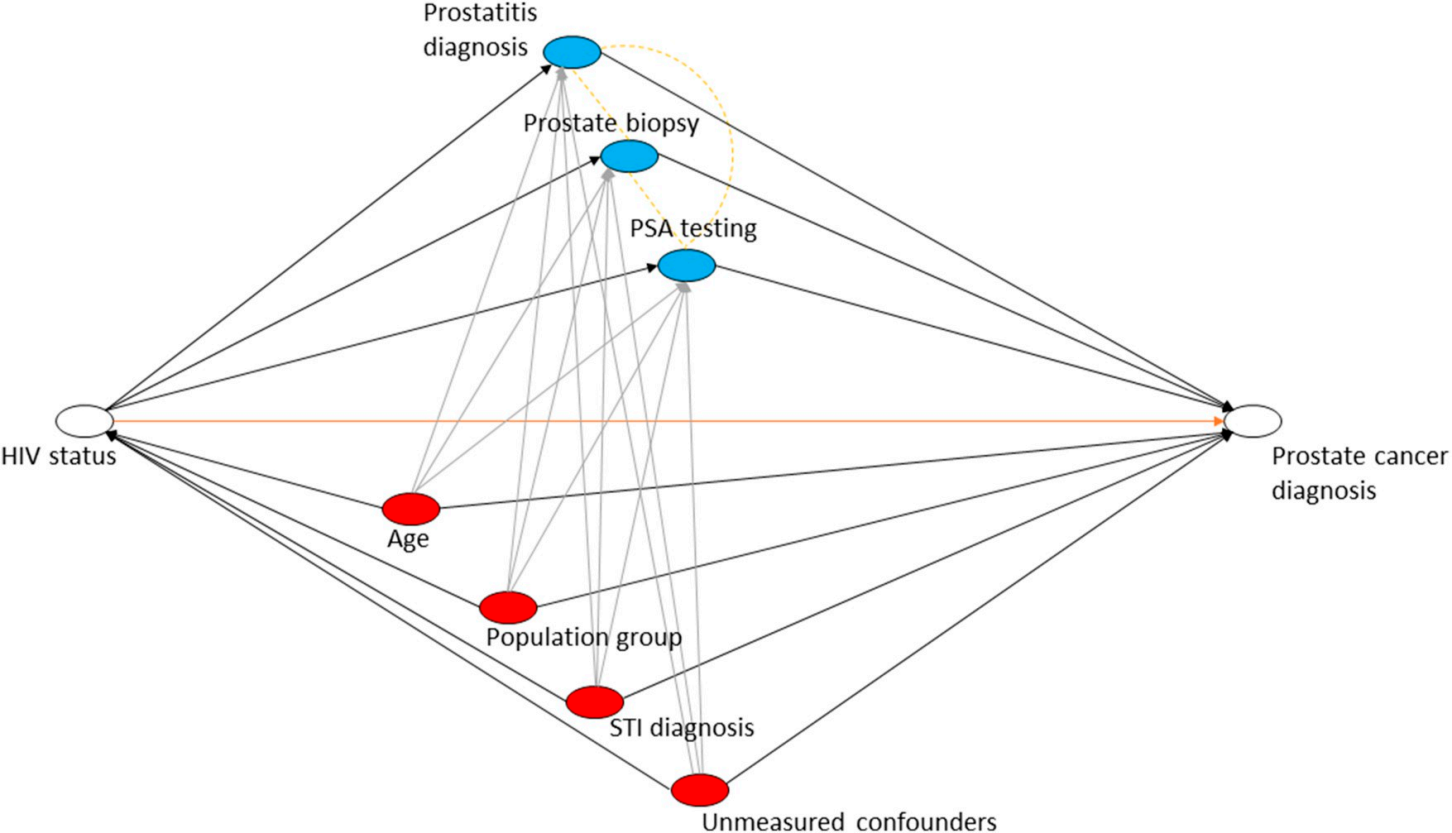
Graph illustrating structural assumptions regarding factors potentially confounding (red nodes) or mediating (blue nodes) the effect of HIV on incident prostate cancer diagnosis. Age, population group, STIs, and other unmeasured factors are assumed to be related with both HIV status and prostate cancer diagnosis and may, therefore, confound the effect of HIV on the risk of incident prostate cancer diagnosis. PSA testing, prostate biopsies, and prostatitis are assumed to be related with HIV status and to affect prostate cancer diagnosis rates and may, thus, serve as mediators. The dashed (yellow) lines indicate influences where the direction is unknown. The (orange) arrow represents the effect of HIV on incident prostate cancer diagnosis, taking into account confounders and mediators. This effect is interpreted as the possible biological effect of HIV on the risk of a prostate cancer diagnosis.

We differentiated between factors that could confound or mediate the effect of HIV on prostate cancer diagnosis rates (Fig. 2). Potential confounders included age (18–39, 40–54, 55–64, 65–74, ≥75 years; time-updated), self-identified population group (black African, white, colored, Indian/Asian, unknown), and a previous STI diagnosis (no/yes; time-updated). Potential mediators included a previous prostatitis diagnosis (no/yes; time-updated), a previous PSA test (no/yes; time-updated), and a previous prostate biopsy (no/yes; time-updated). Colored and Indian/Asian population groups were regrouped into a single category, due to the small number of MWH in these groups.

### Statistical analysis

We performed descriptive data analyses to assess sociodemographic characteristics of included men by HIV status and population group. We calculated crude PSA testing rates, prostate biopsy rates, and prostate cancer diagnosis rates per 100,000 person-years in MWH and men without HIV. When considering PSA tests and prostate biopsies as outcomes, we treated each PSA test and prostate biopsy as a separate event but ignored tests or biopsies that occurred <12 months after a previous PSA test or biopsy. We calculated prostate biopsy rates per 100,000 person-years in men with an elevated PSA test, stratified by HIV status. PSA tests and prostate biopsies occurring on the day of a prostate cancer diagnosis were brought back one day, as they were assumed to have preceded the cancer diagnosis.

We examined the association between HIV and factors potentially mediating its effect on prostate cancer diagnosis rates. We computed rate ratios (RR) for the association between HIV and PSA testing or prostate biopsies using unadjusted and confounder-adjusted Poisson regression with an offset for person-years. Using the same approach, we assessed the association of HIV and prostate biopsies among men with an elevated PSA test result. We used robust estimators to account for clustering of data by individual (18). To estimate hazard ratios (HR) for the association between HIV and incident prostatitis diagnoses, we used unadjusted and confounder-adjusted flexible parametric survival models (19). We used flexible parametric survival models to estimate HRs describing the association between HIV and incident prostate cancer diagnoses. We derived HRs from the following models: (i) unadjusted, (ii) adjusted for age, (iii) adjusted for potential confounders, (iv) adjusted for potential confounders and PSA testing, and (v) adjusted for confounders and mediators (to estimate the residual effect of HIV on prostate cancer diagnoses conditional on confounders and mediators). We report summary HRs based on models assuming proportional hazards and time-varying HRs obtained from including an interaction between follow-up time and each factor of interest. We used flexible parametric survival models to estimate prostate cancer diagnosis rates as a continuous function of age, including HIV status as an independent variable and an interaction between HIV status and age. We chose the number of degrees of freedom for the baseline hazards (Supplementary Table S1) and interactions using the Akaike Information Criteria. Analyses were performed using R 4.2.3 (R Foundation for Statistical Computing).

### Sensitivity and subgroup analyses

To assess the impact of our definition of MWH we performed sensitivity analyses in which: (i) we required MWH to be recorded as AfA members and have at least one additional HIV indicator, and (ii) MWH had the same definition of time-at-risk as men without HIV (no left-truncation at the first HIV indicator). In subgroup analyses, we assessed factors associated with incident prostate cancer diagnoses among (i) men with at least one PSA test, left-truncating follow-up at the first PSA test, (ii) men with an elevated PSA test followed by a prostate biopsy, left-truncating follow-up at the biopsy, and (iii) men from specific age groups. Finally, we changed our definition of a prostate cancer diagnosis to require only one C61 ICD-10 code.

### Data availability

Data were obtained from the International epidemiology Databases to Evaluate AIDS–Southern Africa, and for inquiries about the data, readers can contact them through the online form available at https://www.iedea-sa.org/contact-us/. Further information is available from the corresponding author upon request.

## Results

### Study population

Of 436,294 men covered by the medical insurance scheme at some point between July 1, 2017 and July 1, 2020, we excluded 136,615 men because their follow-up ended before the age of 18 years and another 11,485 men for reasons detailed in Fig. 1. We included 288,194 men in our analysis, of whom 7% were MWH (*n* = 20,074; Table 1). The total time-at-risk was 601,542 years for a median of 2.8 years [interquartile range (IQR) 1.1–3.0) per person. Among the included MWH, 98% had at least one HIV-related ICD-10 code, 89% had at least one ART record, 87% were enrolled in the AfA program, and 69% had at least one HIV-related laboratory record. The median age at the start of time-at-risk was 44.8 years (IQR 38.2–52.3) in MWH and 40.3 years (IQR 29.8–53.8) in men without HIV. About 10% of MWH (*n* = 1,986) and 8% of men without HIV (*n* = 20,553) had at least one PSA test documented during their follow-up time. A history of STIs was more frequently reported for MWH (14%; *n* = 2,755) than men without HIV (4%; *n* = 9,874). In both MWH and men without HIV, the most common specified STI diagnoses were gonococcal infections followed by anogenital herpes simplex and *Chlamydia trachomatis* infections. Median age at start of time-at-risk was highest in white men (46.8 years; IQR 32.0–60.0) and lowest in black African men (38.2 years; IQR 30.2–48.2), see Supplementary Table S2.

**Table 1.** Cohort characteristics by HIV status and overall.

Characteristics	Men without HIV *n* (%)	Men with HIV *n* (%)
Total	268,120	20,074
Median age^a^ (years; IQR)	40.3 (29.8, 53.8)	44.8 (38.2, 52.3)
Age category^a^ (years)		
18–24	44,384 (16.6)	476 (2.4)
25–34	57,046 (21.3)	2,661 (13.3)
35–44	58,091 (21.7)	7,038 (35.1)
45–54	47,194 (17.6)	6,427 (32.0)
55–64	37,198 (13.9)	3,049 (15.2)
65–74	16,132 (6.0)	385 (1.9)
≥75	8,075 (3.0)	38 (0.2)
Calendar year^a^		
2017–2018	227,772 (85.0)	17,097 (85.2)
2019–2020	40,348 (15.0)	2,977 (14.8)
Population group		
Black African	127,900 (47.7)	17,362 (86.5)
Colored	18,186 (6.8)	287 (1.4)
White	53,383 (19.9)	368 (1.8)
Indian/Asian	14,152 (5.3)	122 (0.6)
Unknown	54,499 (20.3)	1,935 (9.6)
PSA test^b^	20,553 (7.7)	1,986 (9.9)
Prostate biopsy^b^	3,657 (1.4)	207 (1.0)
Prostatitis diagnosis^b^	11,235 (4.2)	1,047 (5.2)
STI diagnosis^b^	9,874 (3.7)	2,755 (13.7)

a At start of time-at-risk.

b During or before follow-up.

### PSA testing, prostate biopsies, and prostatitis diagnoses

The crude PSA testing rate was 4,595/100,000 person-years [95% confidence interval (CI), 4,541–4,649], with higher rates in MWH (5,346; 95% CI, 5,130–5,568) than men without HIV (4,536; 95% CI, 4,480–4,593). In the unadjusted and adjusted analyses, MWH were more likely to undergo PSA testing than men without HIV (Table 2), with the association being weaker in the unadjusted analysis (RR 1.18; 95% CI, 1.12–1.24) than the confounder-adjusted analysis (RR 2.24; 95% CI, 2.13–2.36). White men (adjusted RR 2.59; 95% CI, 2.49–2.69) and men of other population groups (adjusted RR 2.01; 95% CI, 1.91–2.11) were more likely to undergo PSA testing than black African men. PSA testing rates were the highest in men aged ≥65 years. PSA levels increased with older age in MWH and men without HIV (Supplementary Table S3).

**Table 2.** Rate or HR for PSA testing, prostate biopsies, prostate biopsies in men with elevated PSA, and prostatitis diagnosis.

Risk factors	RR^a^ (95% CI) for PSA testing		RR^a^ (95% CI) for prostate biopsy		RR^a^ (95% CI) for prostate biopsy in men with elevated PSA		HR^b^ (95% CI) for prostatitis diagnosis	
	Unadjusted	Confounder-adjusted	Unadjusted	Confounder-adjusted	Unadjusted	Confounder-adjusted	Unadjusted	Confounder-adjusted
HIV status								
Negative	1	1	1	1	1	1	1	1
Positive	1.18 (1.12–1.24)	2.24 (2.13–2.36)	0.70 (0.58–0.83)	0.99 (0.82–1.20)	1.39 (0.99–1.95)	0.86 (0.59–1.26)	0.99 (0.88–1.11)	1.18 (1.04–1.32)
Current age								
18–54	0.20 (0.19–0.21)	0.22 (0.21–0.23)	0.07 (0.06–0.08)	0.07 (0.06–0.08)	1.08 (0.83–1.41)	1.07 (0.81–1.42)	0.31 (0.29–0.33)	0.31 (0.29–0.33)
55–64	1	1	1	1	1	1	1	1
65–74	1.55 (1.49–1.60)	1.37 (1.32–1.42)	1.83 (1.67–2.01)	1.88 (1.71–2.08)	0.63 (0.52–0.75)	0.66 (0.54–0.80)	1.41 (1.30–1.54)	1.43 (1.31–1.56)
≥75	1.52 (1.46–1.59)	1.30 (1.25–1.36)	1.67 (1.48–1.88)	1.73 (1.53–1.97)	0.36 (0.28–0.45)	0.38 (0.30–0.49)	1.36 (1.22–1.52)	1.38 (1.24–1.55)
Population group								
Black African	1	1	1	1	1	1	1	1
White	3.77 (3.63–3.91)	2.59 (2.49–2.69)	2.18 (1.96–2.42)	0.88 (0.78–0.99)	0.60 (0.49–0.74)	0.78 (0.63–0.98)	1.68 (1.56–1.81)	1.13 (1.04–1.23)
Colored/Indian/Asian	2.08 (1.98–2.18)	2.01 (1.91–2.11)	1.25 (1.08–1.45)	0.87 (0.75–1.02)	0.74 (0.55–1.01)	0.83 (0.61–1.14)	1.11 (1.00–1.23)	1.00 (0.90–1.11)
Unknown	2.88 (2.78–2.99)	1.87 (1.80–1.95)	2.28 (2.07–2.52)	0.88 (0.79–0.99)	0.58 (0.47–0.71)	0.76 (0.61–0.95)	1.42 (1.32–1.53)	0.92 (0.84–0.99)
STI diagnosis								
No	1	1	1	1	1	1	1	1
Yes	0.52 (0.47–0.57)	0.93 (0.85–1.02)	0.46 (0.35–0.62)	0.85 (0.64–1.14)	1.35 (0.84–2.17)	0.93 (0.56–1.54)	0.90 (0.77–1.05)	1.20 (1.02–1.40)

Confounder-adjusted models include HIV status, current age, population group, and history of STI.

a From Poisson regression.

b From flexible parametric survival models.

In contrast to PSA testing, crude prostate biopsy rates per 100,000 person-years were lower in MWH (288; 95% CI, 240–343) than men without HIV (414; 95% CI, 397–431). In the unadjusted analysis, MWH had lower prostate biopsy rates than men without HIV (RR 0.70; 95% CI, 0.58–0.83), but this association disappeared in the confounder-adjusted analysis (RR 0.99; 95% CI, 0.82–1.20). White men were slightly less likely to undergo prostate biopsies than black African men (adjusted RR 0.88; 95% CI, 0.78–0.99), and biopsy rates were highest in the older age groups. We found no evidence of an association between HIV status and prostate biopsy in the analysis restricted to 2,785 men with an elevated PSA result of >4 ng/mL (adjusted RR 0.86; 95% CI, 0.59–1.27). In this population, biopsy rates were the lowest in men aged ≥75 years (Table 2).

Crude prostatitis diagnosis rates in MWH (776/100,000 person-years; 95% CI, 693–865) and men without HIV (784/100,000 person-years; 95% CI, 761–809) were similar. However, in the confounder-adjusted analysis, a positive HIV status was associated with higher prostatitis diagnosis rates (HR, 1.18; 95% CI, 1.04–1.32). Older age and previous STI diagnoses were also associated with increased prostatitis diagnosis rates (Table 2).

### Incident prostate cancer diagnoses

**Figure 3.**
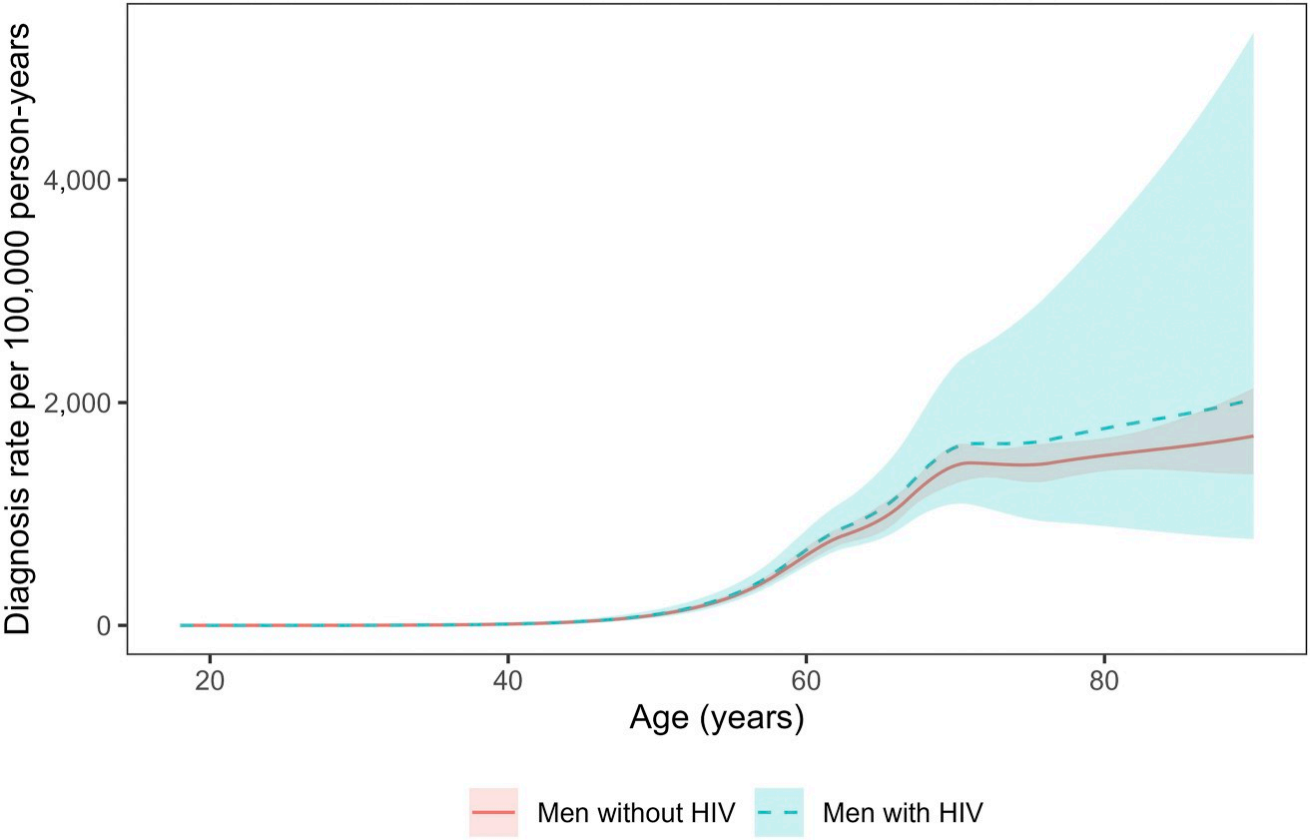
Prostate cancer diagnosis rates by age and HIV status. The figure shows estimated prostate cancer diagnosis rates per 100,000 person-years by age (years) among men with and without HIV. The shaded areas represent 95% CIs.

Incident prostate cancer was diagnosed in 1,614 men without HIV (crude rate: 289/100,000 person-years; 95% CI, 275–304) and in 82 MWH (crude rate: 189/100,000 person-years; 95% CI, 150–235). Median age at prostate cancer diagnosis was higher among men without HIV (67.4 years; IQR: 60.5–73.9) than MWH (59.6 years; IQR 64.9–64.1; Supplementary Table S4). Characteristics at prostate cancer diagnosis by population group are shown in Supplementary Table S5. Age-specific prostate cancer diagnosis rates were similar in MWH and men without HIV, with prostate cancer diagnosis rates increasing steeply between the age of 40 and 70 years (Fig. 3).

In the unadjusted analysis, MWH had a 35% lower risk of incident prostate cancer diagnosis than men without HIV (HR, 0.65; 95% CI, 0.52–0.82; Table 3). This association was no longer evident in the age-adjusted model (HR, 1.10; 95% CI, 0.88–1.38). Further adjusting for additional confounders (HR, 1.03; 95% CI, 0.82–1.30), confounders and PSA testing (HR, 0.95; 95% CI, 0.76–1.20), and both potential confounders and mediators (HR, 1.14; 95% CI, 0.91–1.44) did not change the strength of the association substantially. Supplementary Fig. S1 shows the HRs from the fully adjusted model as a function of follow-up time, relaxing the proportional hazards assumption. The HR for the association between HIV status and incident prostate cancer diagnosis was approximately one during early follow-up and increased thereafter. Characteristics associated with higher prostate cancer diagnosis rates included older age and black African or unknown population group (Table 3).

**Table 3.** Hazard ratios for the association of different factors with an incident prostate cancer diagnosis.

Characteristics	HR (95%CI) unadjusted	HR (95%CI) adjusted for HIV status and age	HR (95%CI) adjusted for potential confounders	HR (95%CI) adjusted for potential confounders and PSA testing	HR (95%CI) adjusted for potential confounders and mediators
HIV status					
Negative	1	1	1	1	1
Positive	0.65 (0.52–0.82)	1.10 (0.88–1.38)	1.03 (0.82–1.30)	0.95 (0.76–1.20)	1.14 (0.91–1.44)
Current age (years)					
18–54	0.06 (0.05–0.08)	0.06 (0.05–0.08)	0.06 (0.05–0.07)	0.07 (0.06–0.09)	0.15 (0.13–0.19)
55–64	1	1	1	1	1
65–74	2.27 (2.03–2.55)	2.28 (2.04–2.56)	2.34 (2.08–2.63)	2.11 (1.87–2.37)	1.33 (1.18–1.50)
≥75	2.57 (2.25–2.94)	2.59 (2.27–2.97)	2.65 (2.31–3.05)	2.44 (2.12–2.81)	1.57 (1.36–1.80)
Population group					
Black African	1		1	1	1
White	2.35 (2.07–2.67)		0.82 (0.72–0.95)	0.68 (0.59–0.78)	0.65 (0.57–0.75)
Colored/Indian/Asian	1.10 (0.91–1.34)		0.72 (0.59–0.87)	0.67 (0.55–0.81)	0.63 (0.52–0.77)
Unknown	2.81 (2.49–3.16)		0.94 (0.82–1.07)	0.85 (0.75–0.97)	1.23 (1.08–1.39)
STI diagnosis					
No	1		1	1	1
Yes	0.40 (0.27–0.57)		0.82 (0.57–1.20)	0.85 (0.59–1.24)	0.74 (0.51–1.07)
Prostatitis diagnosis					
No	1				1
Yes	8.22 (7.38–9.17)				0.73 (0.65–0.82)
PSA test					
No	1			1	1
Yes	11.32 (10.17–12.60)			4.39 (3.93–4.89)	1.99 (1.78–2.21)
Prostate biopsy					
No	1				1
Yes	220.5 (199.1–244.2)				89.0 (78.9–100.3)

Potential confounders include age, population group, and history of STI. Potential mediators include diagnosis of prostatitis, PSA test, and prostate biopsy.

### Sensitivity and subgroup analyses

When varying our definition of MWH and their time-at-risk, results remained broadly similar (Supplementary Tables S6–S9). When restricting the analysis to 22,539 men with at least one PSA test, the estimated association between HIV and incident prostate cancer diagnosis remained similar compared with the main analysis (Supplementary Table S10). When we further restricted the analysis to 634 men who had a prostate biopsy after an elevated PSA test, we found a positive association between HIV and incident prostate cancer diagnosis across all models (Supplementary Table S11). In subgroup analyses by age groups, the unadjusted HRs changed from a positive association of HIV and incident prostate cancer among the youngest age group to a negative association among men aged ≥75 years (Supplementary Table S12). However, the corresponding 95% CIs were wide, and in the fully adjusted models, differences between subgroups were attenuated. Relaxing the definition of a prostate cancer diagnosis to require only one C61 ICD-10 code led to a positive association between HIV and incident prostate cancer diagnosis in the fully adjusted model (HR, 1.25; 95% CI, 1.03–1.51; Supplementary Table S13).

## Discussion

In this analysis of claims data from a medical insurance scheme in South Africa, we found lower crude prostate cancer diagnosis rates among MWH than men without HIV. However, when taking into account potential confounders, the effect of HIV on prostate cancer diagnosis rates was minimal. Other factors, such as age and population group, were more strongly associated with incident prostate cancer diagnoses than HIV. MWH had higher adjusted rates of PSA testing and prostatitis diagnosis but similar prostate biopsy rates than men without HIV. When adjusting for these potential mediators, we found no evidence that HIV led to lower prostate cancer diagnosis rates.

In line with previous US-based studies (5, 6, 20), we found lower crude prostate cancer diagnosis rates among MWH than men without HIV. However, we observed no clear overall effect of HIV on prostate cancer diagnosis rates after adjusting for potential confounders. This contrasts with the results of a 2021 meta-analysis, which found a pooled standardized incidence ratio of 0.76 (95% CI, 0.64–0.91) comparing MWH to men without HIV or the general population, but the heterogeneity between the studies was considerable (1). Of note, all included estimates were adjusted for age, some for race/ethnicity, calendar period, or registry, and one study adjusted for additional factors such as smoking, alcohol or drug abuse, obesity, and diabetes (5). Two of the 27 studies included in this meta-analysis were conducted in sub-Saharan Africa; however, their estimates had very high uncertainty (21, 22). There are few large-scale studies available that directly compared prostate cancer diagnosis rates among MWH and men without HIV. Even fewer studies adjust for potential differences in PSA testing and prostate biopsy patterns by HIV status (5, 6).

It has been hypothesized that lower PSA screening rates among MWH may explain the lower rates of diagnosed prostate cancer among MWH (6, 23). Population-based PSA testing for prostate cancer screening is a controversial topic: the impact of PSA screening on overall mortality appears to be limited, and up to 50% of the detected prostate cancers might not have become clinically relevant within a person’s lifetime (24). The US Preventive Services Task Force recommends individual shared decision-making regarding PSA screening for men aged 55 to 69 years (25). In South Africa, the Council for Medical Schemes recommends PSA screening for men with a life expectancy of ≥10 years from the age of 40 years, if risk factors such as positive family history are present, and from the age of 45 years in all men (26). Prostate cancer screening recommendations generally do not differ by HIV status. Still, early in the HIV epidemic when potent ART was not yet widely available, clinicians may have been reluctant to offer prostate cancer screening to MWH due to poor HIV-related prognosis (4, 27, 28). Two US-based studies found lower PSA testing rates among MWH than men without HIV or the general population (4, 6). In contrast, a study from the Kaiser Permanente integrated health care delivery system in the United States indicated that MWH may be more likely to undergo prostate cancer screening than men without HIV in a managed care setting, potentially due to more regular interactions with the health system (5, 28). In our study, privately insured MWH in South Africa had higher rates of PSA testing than men without HIV.

Interestingly, we and others (6) found lower crude prostate biopsy rates in MWH compared to men without HIV. Prostate biopsies might be performed less frequently among MWH because of the invasive nature of the procedure and the risk of excessive bleeding due to HIV-associated thrombocytopenia (1). In our study, the association between HIV and prostate biopsy rates disappeared after adjusting for confounders or restricting the analysis to men with an elevated PSA. Data on the association between HIV and incident prostatitis diagnoses are limited. Yet, there is some evidence that prostatitis diagnoses are more common among men with AIDS than asymptomatic MWH or the general population (29). In our analysis, MWH were more likely to be diagnosed with prostatitis than men without HIV.

Using models that adjusted for measured confounders and potential mediators, we found no evidence that MWH had lower prostate cancer diagnosis rates than men without HIV. Our findings contrast to some extent with those of the US Veterans Aging Cohort Study, which showed marginally lower prostate cancer rates in MWH than men without HIV when adjusting for potential confounders and PSA testing (incidence rate ratio 0.93; 95% CI, 0.86–1.01; ref. 6). However, when the authors restricted the analysis to men who received prostate biopsies, prostate cancer detection rates were similar in MWH and men without HIV (incidence rate ratio 1.06; 95% CI, 0.98–1.20). Another US-based study found that among men with a previous PSA test, HIV was associated with a lower risk of developing prostate cancer (5). Of note, this study did not consider potential differences in prostate biopsy rates between MWH and men without HIV. The conflicting results may also be explained by differences in the HIV epidemics between countries, the risk factor profiles among the study populations, and the confounders adjusted for. The lower crude prostate cancer diagnosis rates among MWH in our study were mostly due to differences in the age distribution between MWH and men without HIV, with few MWH aged 65 years or older included in our analysis.

Our study is one of few assessing the effect of HIV on prostate cancer diagnosis rates in sub-Saharan Africa. We specifically considered the impact of potential confounders and mediators and included a large sample size. Our study has several limitations. Privately insured men generally have better health care access and different socio-demographic characteristics compared with uninsured men. Thus, our findings are unlikely to be generalizable to the public sector and the general population of South Africa. Furthermore, defining exposures and outcomes based on reimbursement claims data may have led to misclassification and underreporting. However, in sensitivity analyses, we assessed the robustness of our results across different definitions of incident prostate cancer and MWH. We assumed that men without HIV indicators were HIV-negative, but some of these men may have had undiagnosed HIV. Similarly, for STIs and prostatitis, we assumed that men without a corresponding ICD-10 code had no history of STIs or prostatitis. Laboratory data on PSA was only available for the period of 2017 to 2020 and, thus, we restricted the study period to these calendar years. However, information on HIV-related and other medical claims were available from 2011. We were unable to differentiate between PSA tests done for screening purposes and PSA tests done for monitoring purposes, as we had not information on the clinical reasoning behind the PSA tests. However, we excluded PSA tests done after prostate biopsies as monitoring tests. Nevertheless, as symptoms related to undiagnosed prostate cancer may have triggered PSA tests and prostate biopsies, collider bias may have distorted the observed association between HIV and incident prostate cancer diagnosis in the mediator-adjusted models. Moreover, we assumed that misclassification of monitoring PSA tests as screening tests did not differ by HIV status. We were unable to control for behavioral and lifestyle factors that may have distorted our results. Moreover, we were unable to assess differences in advanced stage disease due to a lack of cancer staging information.

In conclusion, we did not find evidence for prostate cancer diagnosis rates to be lower among privately insured men with HIV than men without HIV in South Africa when potential confounders and mediators were considered. Our results do not support the hypothesis that HIV may decrease the risk of prostate cancer through biological mechanisms.

## Supplementary Material

Figure S1 Figure S1 shows hazard ratios for prostate cancer diagnosis over follow-up time.

Table S1 Table S1 shows degrees of freedom chosen for the natural spline bases in the Royston-Parmar flexible parametric survival models.

Table S2 Table S2 shows the cohort characteristics by population group

Table S3 Table S3 shows the median prostate specific antigen (PSA) levels in men with and without HIV.

Table S4 Table S4 shows characteristics at diagnosis of prostate cancer, by HIV status and overall.

Table S5 Table S5 shows characteristics at diagnosis of prostate cancer, by population group.

Table S6 Table S6 shows rate ratios for prostate specific antigen (PSA) testing and prostate biopsies, excluding HIV positive men not in Aid for AIDS registration.

Table S7 Table S7 shows rate ratios for prostate specific antigen testing (PSA) and prostate biopsies, with no left-truncation at first HIV positive marker.

Table S8 Table S8 shows hazard ratios for prostate cancer diagnosis, excluding men with HIV without Aid for AIDS registration.

Table S9 Table S9 shows hazard ratios for prostate cancer diagnosis, with no left-truncation at first HIV indicator.

Table S10 Table S10 shows hazard ratios for prostate cancer diagnosis, restricting to men with at least one prostate specific antigen test.

Table S11 Table S11 shows hazard ratios for prostate cancer diagnosis, restricting to men with an elevated prostate specific antigen test result (>4 ng/mL) followed by a prostate biopsy.

Table S12 Table S12 shows hazard ratios for incident prostate cancer diagnosis among men with HIV compared to men without HIV.

Table S13 Table S13 shows hazard ratios for the association of different factors with an incident prostate cancer diagnosis, including men with a single C61 ICD-10 code.
