# Supplementary material for: Prostate Cancer Diagnosis Rates among Insured Men with and without HIV in South Africa: A Cohort Study
Source: Cancer Epidemiol Biomarkers Prev. 2024 May 7;33(8):1057–64. doi: 10.1158/1055-9965.EPI-24-0137 (PMC11292191; doi:10.1158/1055-9965.EPI-24-0137)
Supplement: Figure S1 — shows hazard ratios for prostate cancer diagnosis over follow-up time. [file epi-24-0137_figure_s1_suppsf1.docx]

**Supplementary Figure 1: Hazard ratios for prostate cancer diagnosis over follow-up time.**


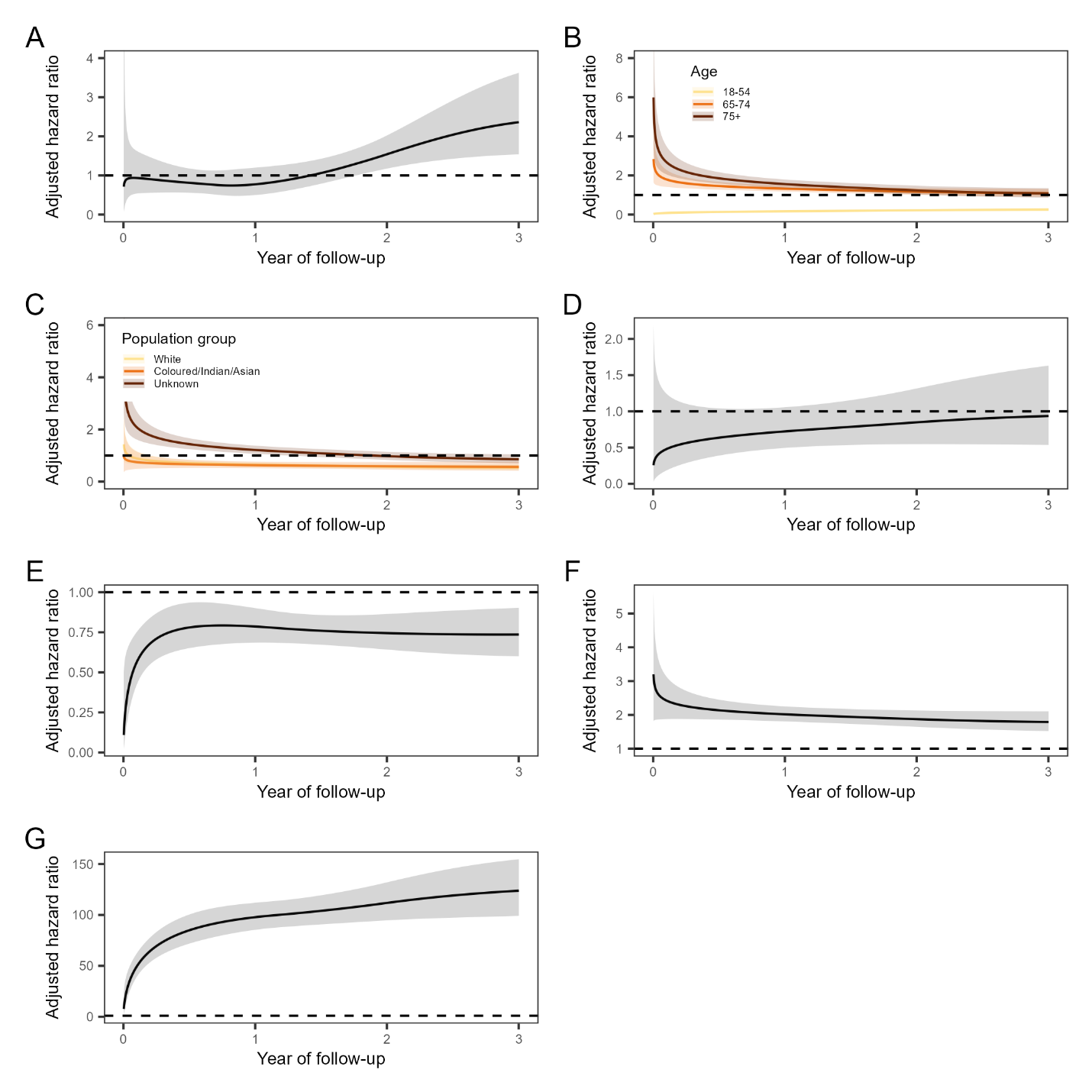


For these analyses we relaxed the proportional hazards assumption by allowing the hazard ratio to change over follow-up time. (A) Men with HIV vs men without HIV; (B) Age group (reference = 55-64 years); (C) Population group (reference = black African); (D) STI diagnosis vs no STI diagnosis; (E) Prostate biopsy vs no biopsy; (F) PSA test vs no PSA test; (G) Prostatitis diagnosis vs no prostatitis diagnosis. Time 0 correspond to the start of the person’s time-at-risk.
