# Supplementary material for: Prostate Cancer Diagnosis Rates among Insured Men with and without HIV in South Africa: A Cohort Study
Source: Cancer Epidemiol Biomarkers Prev. 2024 May 7;33(8):1057–64. doi: 10.1158/1055-9965.EPI-24-0137 (PMC11292191; doi:10.1158/1055-9965.EPI-24-0137)
Supplement: Table S1 — shows degrees of freedom chosen for the natural spline bases in the Royston-Parmar flexible parametric survival models. [file epi-24-0137_table_s1_suppst1.docx]

**Supplementary Table 1**: **Degrees of freedom chosen for the natural spline bases in the Royston-Parmar flexible parametric survival models.**

| **Analysis on follow-up scale** | **Degrees of freedom** |
| --- | --- |
| Baseline hazard | 4 |
| Interactions with follow-up time: |  |
| - HIV status | 3 |
| - Age | 1 |
| - Population group | 1 |
| - PSA test | 1 |
| - Biopsy | 2 |
| - STI | 1 |
| - Prostatitis diagnosis | 2 |
| **Analysis on age scale** | **Degrees of freedom** |
| Baseline hazard | 6 |
| Age-HIV status interaction | 1 |

PSA: prostate specific antigen; STI: sexually transmitted infection
