# Supplementary material for: Prostate Cancer Diagnosis Rates among Insured Men with and without HIV in South Africa: A Cohort Study
Source: Cancer Epidemiol Biomarkers Prev. 2024 May 7;33(8):1057–64. doi: 10.1158/1055-9965.EPI-24-0137 (PMC11292191; doi:10.1158/1055-9965.EPI-24-0137)
Supplement: Table S2 — shows the cohort characteristics by population group [file epi-24-0137_table_s2_suppst2.docx]

**Supplementary Table 2: Cohort characteristics, by population group.**

| **Characteristics** | **Black African**  **n (%)** | **Coloured**  **n (%)** | **White**  **n (%)** | **Indian/Asian**  **n (%)** | **Unknown**  **n (%)** |
| --- | --- | --- | --- | --- | --- |
| **Total** | 145262 | 18473 | 53751 | 14274 | 56434 |
| **HIV status** |  |  |  |  |  |
| Negative | 127900 (88.0) | 18186 (98.4) | 53383 (99.3) | 14152 (99.1) | 54499 (96.6) |
| Positive | 17362 (12.0) | 287 (1.6) | 368 (0.7) | 122 (0.9) | 1935 (3.4) |
| **Median age*** **(years) [IQR]** | 38.2 [30.2, 48.3] | 38.3 [29.3, 48.1] | 46.8 [32.0, 60.1] | 43.1 [31.3, 56.0] | 47.6 [29.8, 60.7] |
| **Age category*** **(years)** |  |  |  |  |  |
| 18-24 | 20788 (14.3) | 3007 (16.3) | 7682 (14.3) | 2028 (14.2) | 11355 (20.1) |
| 25-34 | 37429 (25.8) | 4488 (24.3) | 8600 (16.0) | 2685 (18.8) | 6505 (11.5) |
| 35-44 | 40554 (27.9) | 5027 (27.2) | 8830 (16.4) | 2999 (21.0) | 7719 (13.7) |
| 45-54 | 26813 (18.5) | 3571 (19.3) | 9901 (18.4) | 2704 (18.9) | 10632 (18.8) |
| 55-64 | 16482 (11.3) | 1769 (9.6) | 9808 (18.2) | 2309 (16.2) | 9879 (17.5) |
| 65-74 | 2554 (1.8) | 487 (2.6) | 5738 (10.7) | 1200 (8.4) | 6538 (11.6) |
| ≥75 | 642 (0.4) | 124 (0.7) | 3192 (5.9) | 349 (2.4) | 3806 (6.7) |
| **Calendar year*** |  |  |  |  |  |
| 2017-2018 | 120667 (83.1) | 15184 (82.2) | 44366 (82.5) | 11620 (81.4) | 53032 (94.0) |
| 2019-2020 | 24595 (16.9) | 3289 (17.8) | 9385 (17.5) | 2654 (18.6) | 3402 (6.0) |
| **PSA test**† | 5775 (4.0) | 1823 (9.9) | 7683 (14.3) | 795 (5.6) | 6463 (11.5) |
| **Prostate biopsy**† | 1202 (0.8) | 186 (1.0) | 1256 (2.3) | 210 (1.5) | 1010 (1.8) |
| **Prostatitis diagnosis**† | 5473 (3.8) | 602 (3.3) | 3378 (6.3) | 668 (4.7) | 2161 (3.8) |
| **STI diagnosis**† | 11198 (7.7) | 321 (1.7) | 315 (0.6) | 103 (0.7) | 692 (1.2) |

* At start of time-at-risk

† During or before follow-up

PSA: prostate specific antigen; STI: sexually transmitted infection
