# Supplementary material for: Prostate Cancer Diagnosis Rates among Insured Men with and without HIV in South Africa: A Cohort Study
Source: Cancer Epidemiol Biomarkers Prev. 2024 May 7;33(8):1057–64. doi: 10.1158/1055-9965.EPI-24-0137 (PMC11292191; doi:10.1158/1055-9965.EPI-24-0137)
Supplement: Table S3 — shows the median prostate specific antigen (PSA) levels in men with and without HIV. [file epi-24-0137_table_s3_suppst3.docx]

**Supplementary Table 3**: **Median prostate specific antigen (PSA) levels in men with and without HIV.**

| **Age group [years]** | **Men without HIV** | **Men with HIV** |
| --- | --- | --- |
| **18-44** | 0.62 [0.43-0.96] | 0.69 [0.46-1.05] |
| **45-54** | 0.74 [0.47-1.22] | 0.78 [0.5-1.33] |
| **55-64** | 1.01 [0.58-1.94] | 0.99 [0.58-1.89] |
| **65-74** | 1.44 [0.77-2.98] | 1.44 [0.68-2.7] |
| **≥75** | 1.95 [0.84-4.12] | 2.10 [1.08-4.12] |

The study population is restricted to men who received a PSA test during their time-at-risk. Interquartile ranges are given in brackets. Only the first PSA test after start of time-at-risk for each individual is taken into account. Data are stratified by age group.
