# Supplementary material for: Prostate Cancer Diagnosis Rates among Insured Men with and without HIV in South Africa: A Cohort Study
Source: Cancer Epidemiol Biomarkers Prev. 2024 May 7;33(8):1057–64. doi: 10.1158/1055-9965.EPI-24-0137 (PMC11292191; doi:10.1158/1055-9965.EPI-24-0137)
Supplement: Table S4 — shows characteristics at diagnosis of prostate cancer, by HIV status and overall. [file epi-24-0137_table_s4_suppst4.docx]

**Supplementary Table 4:** **Characteristics at diagnosis of prostate cancer, by HIV status and overall.**

| **Characteristics** | **Men without HIV**  **n (%)** | **Men with HIV**  **n (%)** |
| --- | --- | --- |
| **Total** | 1 614 | 82 |
| **Median age at diagnosis (years) [IQR]** | 67.4 [60.5, 73.9] | 59.6 [54.9, 64.1] |
| **Age at diagnosis (years)** |  |  |
| 18-34 | 3 (0.2) | 0 (0.0) |
| 35-44 | 17 (1.1) | 2 (2.4) |
| 45-54 | 123 (7.6) | 19 (23.2) |
| 55-64 | 546 (33.8) | 46 (56.1) |
| 65-74 | 582 (36.1) | 14 (17.1) |
| ≥75 | 343 (21.3) | 1 (1.2) |
| **Year of diagnosis** |  |  |
| 2017-2018 | 883 (54.7) | 34 (41.5) |
| 2019-2020 | 731 (45.3) | 48 (58.5) |
| **Population group** |  |  |
| Black African | 435 (27.0) | 69 (84.1) |
| Coloured | 74 (4.6) | 1 (1.2) |
| White | 458 (28.4) | 2 (2.4) |
| Indian/Asian | 53 (3.3) | 0 (0.0) |
| Unknown | 594 (36.8) | 10 (12.2) |
| **PSA test**† | 554 (34.3) | 35 (42.7) |
| **Biopsy**† | 1 068 (66.2) | 63 (76.8) |
| **Prostatitis diagnosis**† | 425 (26.3) | 23 (28.0) |
| **STI diagnosis**† | 23 (1.4) | 6 (7.3) |

* P-values from chi-squared test (categorical variables) and nonormal test (continuous variables) comparing men without HIV to wen with HIV.

† During or before follow-up.
