# Supplementary material for: Prostate Cancer Diagnosis Rates among Insured Men with and without HIV in South Africa: A Cohort Study
Source: Cancer Epidemiol Biomarkers Prev. 2024 May 7;33(8):1057–64. doi: 10.1158/1055-9965.EPI-24-0137 (PMC11292191; doi:10.1158/1055-9965.EPI-24-0137)
Supplement: Table S5 — shows characteristics at diagnosis of prostate cancer, by population group. [file epi-24-0137_table_s5_suppst5.docx]

**Supplementary Table 5:** **Characteristics at diagnosis of prostate cancer, by population group.**

| **Characteristics** | **Black African**  **n (%)** | **Coloured**  **n (%)** | **White**  **n (%)** | **Indian/Asian**  **n (%)** | **Unknown**  **n (%)** |
| --- | --- | --- | --- | --- | --- |
| **Total** | 504 | 75 | 460 | 53 | 604 |
| **HIV status** |  |  |  |  |  |
| Negative | 435 (86.3) | 74 (98.7) | 458 (99.6) | 53 (100.0) | 594 (98.3) |
| Positive | 69 (13.7) | 1 (1.3) | 2 (0.4) | 0 (0.0) | 10 (1.7) |
| **Median age at diagnosis (years) [IQR]** | 61.9 [57.2, 66.9] | 62.6 [57.2, 69.9] | 68.5 [62.5, 74.5] | 68.8 [63.5, 72.5] | 70.3 [63.1, 76.3] |
| **Age at diagnosis (years)** |  |  |  |  |  |
| 18-34 | 3 (0.6) | 0 (0.0) | 0 (0.0) | 0 (0.0) | 0 (0.0) |
| 35-44 | 7 (1.4) | 0 (0.0) | 6 (1.3) | 0 (0.0) | 6 (1.0) |
| 45-54 | 72 (14.3) | 16 (21.3) | 28 (6.1) | 1 (1.9) | 25 (4.1) |
| 55-64 | 261 (51.8) | 30 (40.0) | 131 (28.5) | 15 (28.3) | 155 (25.7) |
| 65-74 | 117 (23.2) | 23 (30.7) | 189 (41.1) | 29 (54.7) | 238 (39.4) |
| ≥75 | 44 (8.7) | 6 (8.0) | 106 (23.0) | 8 (15.1) | 180 (29.8) |
| **Year of diagnosis** |  |  |  |  |  |
| 2017-2018 | 238 (47.2) | 33 (44.0) | 246 (53.5) | 32 (60.4) | 368 (60.9) |
| 2019-2020 | 266 (52.8) | 42 (56.0) | 214 (46.5) | 21 (39.6) | 236 (39.1) |
| **PSA test*** | 159 (31.5) | 39 (52.0) | 193 (42.0) | 9 (17.0) | 189 (31.3) |
| **Prostate Biopsy*** | 368 (73.0) | 56 (74.7) | 308 (67.0) | 38 (71.7) | 361 (59.8) |
| **Prostatitis diagnosis*** | 159 (31.5) | 21 (28.0) | 129 (28.0) | 19 (35.8) | 120 (19.9) |
| **STI diagnosis*** | 25 (5.0) | 2 (2.7) | 0 (0.0) | 0 (0.0) | 2 (0.3) |

*During or before follow-up

PSA: prostate specific antigen; STI: sexually transmitted infection
