# Supplementary material for: Prostate Cancer Diagnosis Rates among Insured Men with and without HIV in South Africa: A Cohort Study
Source: Cancer Epidemiol Biomarkers Prev. 2024 May 7;33(8):1057–64. doi: 10.1158/1055-9965.EPI-24-0137 (PMC11292191; doi:10.1158/1055-9965.EPI-24-0137)
Supplement: Table S6 — shows rate ratios for prostate specific antigen (PSA) testing and prostate biopsies, excluding HIV positive men not in Aid for AIDS registration. [file epi-24-0137_table_s6_suppst6.docx]

**Supplementary Table 6:** **Rate ratios for prostate specific antigen (PSA) testing and prostate biopsies, excluding HIV positive men not in Aid for AIDS registration.**

| **Risk Factors** | **RR (95% CI) for PSA testing** | | **RR (95% CI) for prostate biopsy** | |
| --- | --- | --- | --- | --- |
|  | unadjusted | confounder-adjusted | unadjusted | confounder-adjusted |
| **HIV status** |  |  |  |  |
| Negative | 1 | 1 | 1 | 1 |
| Positive | 1.18 (1.12-1.24) | 2.27 (2.15-2.39) | 0.67 (0.55-0.81) | 0.97 (0.79-1.19) |
| **Current age (years)** |  |  |  |  |
| 18-54 | 0.20 (0.19-0.20) | 0.22 (0.21-0.23) | 0.07 (0.06-0.08) | 0.07 (0.06-0.08) |
| 55-64 | 1 | 1 | 1 | 1 |
| 65-74 | 1.55 (1.50-1.60) | 1.37 (1.32-1.42) | 1.84 (1.68-2.03) | 1.90 (1.72-2.09) |
| ≥75 | 1.52 (1.46-1.59) | 1.30 (1.25-1.36) | 1.68 (1.49-1.89) | 1.74 (1.53-1.97) |
| **Population group** |  |  |  |  |
| Black African | 1 | 1 | 1 | 1 |
| White | 3.83 (3.70-3.98) | 2.60 (2.50-2.71) | 2.19 (1.98-2.44) | 0.88 (0.78-0.99) |
| Coloured/Indian/Asian | 2.11 (2.01-2.22) | 2.02 (1.92-2.12) | 1.26 (1.08-1.46) | 0.87 (0.75-1.02) |
| Unknown | 2.93 (2.82-3.05) | 1.88 (1.81-1.96) | 2.30 (2.08-2.55) | 0.89 (0.79-0.99) |
| **History of STI** |  |  |  |  |
| No | 1 | 1 | 1 | 1 |
| Yes | 0.51 (0.46-0.56) | 0.93 (0.85-1.03) | 0.48 (0.36-0.63) | 0.89 (0.67-1.18) |

CI: confidence interval; RR: rate ratio; PSA: prostate specific antigen; STI: sexually transmitted infection
