# Supplementary material for: Prostate Cancer Diagnosis Rates among Insured Men with and without HIV in South Africa: A Cohort Study
Source: Cancer Epidemiol Biomarkers Prev. 2024 May 7;33(8):1057–64. doi: 10.1158/1055-9965.EPI-24-0137 (PMC11292191; doi:10.1158/1055-9965.EPI-24-0137)
Supplement: Table S7 — shows rate ratios for prostate specific antigen testing (PSA) and prostate biopsies, with no left-truncation at first HIV positive marker. [file epi-24-0137_table_s7_suppst7.docx]

**Supplementary Table 7: Rate ratios for prostate specific antigen testing (PSA) and prostate biopsies, with no left-truncation at first HIV positive marker.**

| **Risk factors** | **RR (95% CI) for PSA testing** | | **RR (95% CI) for prostate biopsy** | |
| --- | --- | --- | --- | --- |
|  | **Unadjusted RR (95% CI)** | **Adjusted RR (95% CI)** | **Unadjusted RR (95% CI)** | **Adjusted RR (95% CI)** |
| **HIV status** |  |  |  |  |
| Negative | 1 | 1 | 1 | 1 |
| Positive | 1.10 (1.04-1.15) | 2.11 (2.01-2.22) | 0.68 (0.57-0.81) | 1.00 (0.83-1.19) |
| **Current age (years)** |  |  |  |  |
| 18-54 | 0.20 (0.19-0.20) | 0.22 (0.21-0.23) | 0.07 (0.06-0.08) | 0.07 (0.06-0.08) |
| 55-64 | 1 | 1 | 1 | 1 |
| 65-74 | 1.55 (1.50-1.60) | 1.37 (1.32-1.42) | 1.84 (1.67-2.02) | 1.89 (1.71-2.08) |
| ≥75 | 1.53 (1.46-1.59) | 1.30 (1.25-1.36) | 1.67 (1.48-1.88) | 1.73 (1.53-1.97) |
| **Population group** |  |  |  |  |
| Black African | 1 | 1 | 1 | 1 |
| White | 3.79 (3.66-3.93) | 2.59 (2.49-2.69) | 2.19 (1.98-2.43) | 0.88 (0.78-0.99) |
| Coloured/Indian/Asian | 2.09 (1.99-2.20) | 2.01 (1.91-2.11) | 1.25 (1.08-1.46) | 0.87 (0.75-1.02) |
| Unknown | 2.90 (2.79-3.01) | 1.87 (1.80-1.95) | 2.29 (2.08-2.53) | 0.88 (0.79-0.99) |
| **STI diagnosis** |  |  |  |  |
| No | 1 | 1 | 1 | 1 |
| Yes | 0.51 (0.47-0.56) | 0.94 (0.86-1.03) | 0.46 (0.34-0.61) | 0.84 (0.63-1.12) |

CI: confidence interval; RR: rate ratio; PSA: prostate specific antigen; STI: sexually transmitted infection
