# Supplementary material for: Prostate Cancer Diagnosis Rates among Insured Men with and without HIV in South Africa: A Cohort Study
Source: Cancer Epidemiol Biomarkers Prev. 2024 May 7;33(8):1057–64. doi: 10.1158/1055-9965.EPI-24-0137 (PMC11292191; doi:10.1158/1055-9965.EPI-24-0137)
Supplement: Table S8 — shows hazard ratios for prostate cancer diagnosis, excluding men with HIV without Aid for AIDS registration. [file epi-24-0137_table_s8_suppst8.docx]

**Supplementary Table 8:** **Hazard ratios for prostate cancer diagnosis, excluding men with HIV without Aid for AIDS registration.**

| **Characteristics** | **HR (95% CI)**  unadjusted | **HR (95% CI)**  adjusted for HIV status and age | **HR (95% CI)**  adjusted for potential confounders | **HR (95% CI)**  adjusted for potential confounders and PSA testing | **HR (95% CI)**  adjusted for potential confounders and mediators |
| --- | --- | --- | --- | --- | --- |
| **HIV status** |  |  |  |  |  |
| Negative | 1 | 1 | 1 | 1 | 1 |
| Positive | 0.62 (0.49-0.79) | 1.07 (0.84-1.36) | 1.00 (0.78-1.29) | 0.94 (0.73-1.20) | 1.15 (0.89-1.47) |
| **Current age (years)** |  |  |  |  |  |
| 18-54 | 0.06 (0.05-0.08) | 0.06 (0.05-0.08) | 0.06 (0.05-0.07) | 0.07 (0.06-0.09) | 0.15 (0.13-0.18) |
| 55-64 | 1 | 1 | 1 | 1 | 1 |
| 65-74 | 2.29 (2.04-2.57) | 2.30 (2.05-2.58) | 2.35 (2.09-2.65) | 2.12 (1.88-2.39) | 1.34 (1.19-1.51) |
| ≥75 | 2.59 (2.27-2.96) | 2.60 (2.28-2.98) | 2.66 (2.31-3.06) | 2.45 (2.13-2.82) | 1.57 (1.36-1.80) |
| **Population group** |  |  |  |  |  |
| Black African | 1 |  | 1 | 1 | 1 |
| White | 2.38 (2.10-2.71) |  | 0.83 (0.72-0.95) | 0.68 (0.59-0.78) | 0.66 (0.57-0.75) |
| Coloured/Indian/Asian | 1.12 (0.92-1.35) |  | 0.72 (0.59-0.88) | 0.67 (0.55-0.82) | 0.64 (0.52-0.78) |
| Unknown | 2.85 (2.53-3.21) |  | 0.94 (0.83-1.07) | 0.86 (0.75-0.98) | 1.24 (1.09-1.41) |
| **STI diagnosis** |  |  |  |  |  |
| No | 1 |  | 1 | 1 | 1 |
| Yes | 0.39 (0.27-0.57) |  | 0.83 (0.57-1.22) | 0.86 (0.59-1.26) | 0.76 (0.52-1.11) |
| **Prostatitis diagnosis** |  |  |  |  |  |
| No | 1 |  |  |  | 1 |
| Yes | 8.32 (7.46-9.28) |  |  |  | 0.74 (0.66-0.84) |
| **PSA test** |  |  |  |  |  |
| No | 1 |  |  | 1 | 1 |
| Yes | 11.30 (10.14-12.58) |  |  | 4.36 (3.91-4.87) | 1.98 (1.78-2.21) |
| **Prostate biopsy** |  |  |  |  |  |
| No | 1 |  |  |  | 1 |
| Yes | 219.31 (197.98-242.94) |  |  |  | 87.79 (77.87-98.98) |

CI: confidence interval; HR: hazard ratio; PSA: prostate specific antigen; STI: sexually transmitted infection

Potential confounders include age, population group, and diagnosis of sexually transmitted infection. Potential mediators include diagnosis of prostatitis, prostate specific antigen test, and prostate biopsy.
