# Supplementary material for: Prostate Cancer Diagnosis Rates among Insured Men with and without HIV in South Africa: A Cohort Study
Source: Cancer Epidemiol Biomarkers Prev. 2024 May 7;33(8):1057–64. doi: 10.1158/1055-9965.EPI-24-0137 (PMC11292191; doi:10.1158/1055-9965.EPI-24-0137)
Supplement: Table S9 — shows hazard ratios for prostate cancer diagnosis, with no left-truncation at first HIV indicator. [file epi-24-0137_table_s9_suppst9.docx]

**Supplementary Table 9:** **Hazard ratios for prostate cancer diagnosis, with no left-truncation at first HIV indicator.**

| **Characteristics** | **HR (95% CI)**  unadjusted | **HR (95% CI)**  adjusted for HIV status and age | **HR (95% CI)**  adjusted for potential confounders | **HR (95% CI)**  adjusted for potential confounders and PSA testing | **HR (95% CI)**  adjusted for potential confounders and mediators |
| --- | --- | --- | --- | --- | --- |
| **HIV status** |  |  |  |  |  |
| Negative | 1 | 1 | 1 | 1 | 1 |
| Positive | 0.66 (0.54-0.82) | 1.16 (0.94-1.43) | 1.09 (0.88-1.36) | 1.03 (0.83-1.29) | 1.24 (0.99-1.54) |
| **Current age (years)** |  |  |  |  |  |
| 18-54 | 0.06 (0.05-0.08) | 0.06 (0.05-0.08) | 0.06 (0.05-0.08) | 0.07 (0.06-0.09) | 0.16 (0.13-0.19) |
| 55-64 | 1 | 1 | 1 | 1 | 1 |
| 65-74 | 2.27 (2.03-2.54) | 2.29 (2.04-2.57) | 2.34 (2.08-2.63) | 2.10 (1.87-2.37) | 1.33 (1.18-1.49) |
| ≥75 | 2.56 (2.25-2.93) | 2.60 (2.27-2.97) | 2.65 (2.30-3.05) | 2.44 (2.12-2.80) | 1.56 (1.36-1.79) |
| **Population group** |  |  |  |  |  |
| Black African | 1 |  | 1 | 1 | 1 |
| White | 2.35 (2.07-2.67) |  | 0.83 (0.72-0.95) | 0.68 (0.59-0.78) | 0.65 (0.57-0.75) |
| Coloured/Indian/Asian | 1.10 (0.90-1.33) |  | 0.72 (0.59-0.88) | 0.67 (0.55-0.81) | 0.63 (0.52-0.77) |
| Unknown | 2.82 (2.50-3.17) |  | 0.94 (0.83-1.08) | 0.86 (0.76-0.98) | 1.23 (1.09-1.40) |
| **STI diagnosis** |  |  |  |  |  |
| No | 1 |  | 1 | 1 | 1 |
| Yes | 0.39 (0.27-0.56) |  | 0.80 (0.55-1.17) | 0.83 (0.57-1.21) | 0.72 (0.50-1.05) |
| **Prostatitis diagnosis** |  |  |  |  |  |
| No | 1 |  |  |  | 1 |
| Yes | 8.21 (7.36-9.15) |  |  |  | 0.72 (0.64-0.81) |
| **PSA test** |  |  |  |  |  |
| No | 1 |  |  | 1 | 1 |
| Yes | 11.40 (10.25-12.69) |  |  | 4.42 (3.96-4.93) | 1.99 (1.79-2.22) |
| **Prostate biopsy** |  |  |  |  |  |
| No | 1 |  |  |  | 1 |
| Yes | 222.66 (201.12-246.49) |  |  |  | 90.49 (80.30-101.97) |

CI: confidence interval; HR: hazard ratio; PSA: prostate specific antigen; STI: sexually transmitted infection

Potential confounders include age, population group, and diagnosis of sexually transmitted infection. Potential mediators include diagnosis of prostatitis, prostate specific antigen test, and prostate biopsy.
