# Supplementary material for: Prostate Cancer Diagnosis Rates among Insured Men with and without HIV in South Africa: A Cohort Study
Source: Cancer Epidemiol Biomarkers Prev. 2024 May 7;33(8):1057–64. doi: 10.1158/1055-9965.EPI-24-0137 (PMC11292191; doi:10.1158/1055-9965.EPI-24-0137)
Supplement: Table S10 — shows hazard ratios for prostate cancer diagnosis, restricting to men with at least one prostate specific antigen test. [file epi-24-0137_table_s10_suppst10.docx]

**Supplementary Table 10: Hazard ratios for prostate cancer diagnosis, restricting to men with at least one prostate specific antigen test.**

| **Characteristics** | **HR (95% CI)**  unadjusted | **HR (95% CI)**  adjusted for potential confounders | **HR (95% CI)**  adjusted for potential confounders and mediators |
| --- | --- | --- | --- |
| **HIV status** |  |  |  |
| Negative | 1 | 1 | 1 |
| Positive | 0.70 (0.50-0.98) | 0.83 (0.58-1.20) | 1.19 (0.82-1.71) |
| **Current age (years)** |  |  |  |
| 18-54 | 0.30 (0.22-0.41) | 0.29 (0.21-0.39) | 0.55 (0.41-0.76) |
| 55-64 | 1 | 1 | 1 |
| 65-74 | 1.58 (1.30-1.91) | 1.75 (1.44-2.13) | 1.13 (0.93-1.37) |
| ≥75 | 1.67 (1.33-2.10) | 1.93 (1.52-2.45) | 1.43 (1.13-1.81) |
| **Population group** |  |  |  |
| Black African | 1 | 1 | 1 |
| White | 0.84 (0.68-1.04) | 0.53 (0.42-0.67) | 0.61 (0.49-0.76) |
| Coloured/Indian/Asian | 0.64 (0.46-0.88) | 0.57 (0.41-0.79) | 0.67 (0.48-0.93) |
| Unknown | 0.99 (0.80-1.22) | 0.60 (0.48-0.76) | 0.96 (0.77-1.20) |
| **STI diagnosis** |  |  |  |
| No | 1 | 1 | 1 |
| Yes | 0.95 (0.55-1.65) | 1.17 (0.67-2.06) | 0.86 (0.49-1.51) |
| **Prostatitis diagnosis** |  |  |  |
| No | 1 |  | 1 |
| Yes | 3.07 (2.58-3.65) |  | 0.77 (0.64-0.92) |
| **Prostate biopsy** |  |  |  |
| No | 1 |  | 1 |
| Yes | 67.82 (55.90-82.28) |  | 67.44 (54.93-82.79) |

CI, confidence interval; HR, hazard ratio; STI, sexually transmitted infection

Potential confounders include age, population group, and diagnosis of sexually transmitted infection. Potential mediators include diagnosis of prostatitis and prostate biopsy.
