# Supplementary material for: Prostate Cancer Diagnosis Rates among Insured Men with and without HIV in South Africa: A Cohort Study
Source: Cancer Epidemiol Biomarkers Prev. 2024 May 7;33(8):1057–64. doi: 10.1158/1055-9965.EPI-24-0137 (PMC11292191; doi:10.1158/1055-9965.EPI-24-0137)
Supplement: Table S11 — shows hazard ratios for prostate cancer diagnosis, restricting to men with an elevated prostate specific antigen test result (>4 ng/mL) followed by a prostate biopsy. [file epi-24-0137_table_s11_suppst11.docx]

**Supplementary Table 11: Hazard ratios for prostate cancer diagnosis, restricting to men with an elevated prostate specific antigen test result (>4 ng/mL) followed by a prostate biopsy.**

| **Characteristics** | **HR (95% CI)**  unadjusted | **HR (95% CI)**  adjusted for potential confounders | **HR (95% CI)**  adjusted for potential confounders and mediators |
| --- | --- | --- | --- |
| **HIV status** |  |  |  |
| Negative | 1 | 1 | 1 |
| Positive | 1.45 (0.95-2.22) | 1.55 (0.99-2.45) | 1.52 (0.96-2.40) |
| **Current age (years)** |  |  |  |
| 18-54 | 0.94 (0.64-1.38) | 0.88 (0.60-1.30) | 0.86 (0.58-1.27) |
| 55-64 | 1 | 1 | 1 |
| 65-74 | 1.04 (0.81-1.34) | 1.10 (0.85-1.43) | 1.09 (0.84-1.41) |
| ≥75 | 1.00 (0.72-1.39) | 1.07 (0.76-1.49) | 1.04 (0.75-1.46) |
| **Population group** |  |  |  |
| Black African | 1 | 1 | 1 |
| White | 0.98 (0.74-1.29) | 1.02 (0.76-1.36) | 1.01 (0.75-1.35) |
| Coloured/Indian/Asian | 1.28 (0.85-1.93) | 1.34 (0.89-2.02) | 1.29 (0.86-1.95) |
| Unknown | 0.88 (0.66-1.18) | 0.91 (0.67-1.22) | 0.85 (0.63-1.16) |
| **STI diagnosis** |  |  |  |
| No | 1 | 1 | 1 |
| Yes | 1.19 (0.64-2.24) | 1.21 (0.64-2.30) | 1.23 (0.65-2.33) |
| **Prostatitis diagnosis** |  |  |  |
| No | 1 |  | 1 |
| Yes | 0.77 (0.61-0.97) |  | 0.75 (0.59-0.95) |

CI, confidence interval; HR, hazard ratio; STI, sexually transmitted infection

Potential confounders include age, population group, and diagnosis of sexually transmitted infection. Potential mediators include diagnosis of prostatitis.
