# Supplementary material for: Prostate Cancer Diagnosis Rates among Insured Men with and without HIV in South Africa: A Cohort Study
Source: Cancer Epidemiol Biomarkers Prev. 2024 May 7;33(8):1057–64. doi: 10.1158/1055-9965.EPI-24-0137 (PMC11292191; doi:10.1158/1055-9965.EPI-24-0137)
Supplement: Table S12 — shows hazard ratios for incident prostate cancer diagnosis among men with HIV compared to men without HIV. [file epi-24-0137_table_s12_suppst12.docx]

**Supplemental Table 12. Hazard ratios for incident prostate cancer diagnosis among men with HIV compared to men without HIV.**

| **Age group [years]** | **HR (95% CI)**  unadjusted | **HR (95% CI)**  adjusted for potential confounders | **HR (95% CI)**  adjusted for potential confounders and PSA testing | **HR (95% CI)**  adjusted for potential confounders and mediators |
| --- | --- | --- | --- | --- |
| **18-54** | 1.69 (1.07-2.67) | 1.93 (1.20-3.12) | 1.23 (0.74-2.02) | 1.03 (0.64-1.67) |
| **55-64** | 1.01 (0.75-1.37) | 0.96 (0.70-1.31) | 0.87 (0.64-1.20) | 1.18 (0.86-1.61) |
| **65-74** | 0.94 (0.55-1.60) | 0.82 (0.48-1.41) | 0.76 (0.44-1.30) | 0.83 (0.48-1.43) |
| **≥75** | 0.63 (0.09-4.48) | 0.51 (0.07-3.69) | 0.48 (0.07-3.44) | 0.49 (0.07-3.55) |

CI, confidence interval; HR, hazard ratio; PSA, prostate specific antigen

Data are stratified by age group. Potential confounders include population group and history of sexually transmitted infection. Potential mediators include diagnosis of prostatitis, prostate specific antigen test, and prostate biopsy.
