# Supplementary material for: Prostate Cancer Diagnosis Rates among Insured Men with and without HIV in South Africa: A Cohort Study
Source: Cancer Epidemiol Biomarkers Prev. 2024 May 7;33(8):1057–64. doi: 10.1158/1055-9965.EPI-24-0137 (PMC11292191; doi:10.1158/1055-9965.EPI-24-0137)
Supplement: Table S13 — shows hazard ratios for the association of different factors with an incident prostate cancer diagnosis, including men with a single C61 ICD-10 code. [file epi-24-0137_table_s13_suppst13.docx]

**Supplemental Table 13.** **Hazard ratios for the association of different factors with an incident prostate cancer diagnosis, including men with a single C61 ICD-10 code.**

| **Characteristics** | **HR (95% CI)**  unadjusted | **HR (95% CI)**  adjusted for HIV status and age | **HR (95% CI)**  adjusted for potential confounders | **HR (95% CI)**  adjusted for potential confounders and PSA testing | **HR (95% CI)**  adjusted for potential confounders and mediators |
| --- | --- | --- | --- | --- | --- |
| **HIV status** |  |  |  |  |  |
| Negative | 1 | 1 | 1 | 1 | 1 |
| Positive | 0.70 (0.59-0.85) | 1.22 (1.01-1.47) | 1.15 (0.94-1.39) | 1.06 (0.88-1.29) | 1.25 (1.03-1.51) |
| **Current age (years)** |  |  |  |  |  |
| 18-54 | 0.08 (0.07-0.09) | 0.08 (0.07-0.09) | 0.08 (0.07-0.09) | 0.09 (0.08-0.11) | 0.15 (0.13-0.18) |
| 55-64 | 1 | 1 | 1 | 1 | 1 |
| 65-74 | 2.32 (2.10-2.57) | 2.35 (2.12-2.60) | 2.39 (2.15-2.65) | 2.16 (1.94-2.40) | 1.47 (1.32-1.63) |
| ≥75 | 3.08 (2.75-3.44) | 3.12 (2.79-3.50) | 3.16 (2.81-3.56) | 2.92 (2.59-3.29) | 2.02 (1.80-2.27) |
| **Population group** |  |  |  |  |  |
| Black African | 1 |  | 1 | 1 | 1 |
| White | 2.35 (2.11-2.62) |  | 0.84 (0.74-0.94) | 0.70 (0.62-0.79) | 0.68 (0.60-0.77) |
| Coloured/Indian/Asian | 1.07 (0.90-1.26) |  | 0.71 (0.60-0.85) | 0.67 (0.56-0.79) | 0.64 (0.54-0.77) |
| Unknown | 2.93 (2.64-3.24) |  | 0.99 (0.88-1.10) | 0.91 (0.81-1.02) | 1.25 (1.12-1.39) |
| **STI diagnosis** |  |  |  |  |  |
| No | 1 |  | 1 | 1 | 1 |
| Yes | 0.45 (0.34-0.61) |  | 0.94 (0.69-1.27) | 0.97 (0.72-1.31) | 0.86 (0.64-1.16) |
| **Prostatitis diagnosis** |  |  |  |  |  |
| No | 1 |  |  |  | 1 |
| Yes | 7.08 (6.43-7.80) |  |  |  | 0.78 (0.71-0.87) |
| **PSA test** |  |  |  |  |  |
| No | 1 |  |  | 1 | 1 |
| Yes | 10.27 (9.37-11.27) |  |  | 4.05 (3.68-4.45) | 2.12 (1.93-2.33) |
| **Prostate biopsy** |  |  |  |  |  |
| No | 1 |  |  |  | 1 |
| Yes | 131.60 (121.08-143.04) |  |  |  | 48.97 (44.39-54.02) |

CI, confidence interval; HR, hazard ratio; PSA, prostate specific antigen; STI, sexually transmitted infection.

Potential confounders include age, population group, and history of sexually transmitted infection. Potential mediators include diagnosis of prostatitis, prostate specific antigen test, and prostate biopsy.
